# The predictive role of right ventricular late gadolinium enhancement in patients with tetralogy of Fallot undergoing pulmonary valve replacement

**DOI:** 10.1186/s41747-023-00322-3

**Published:** 2023-02-24

**Authors:** Caterina Beatrice Monti, Moreno Zanardo, Davide Capra, Giulia Lastella, Gianluca Guarnieri, Emilia Giambersio, Giulia Pasqualin, Francesco Sardanelli, Francesco Secchi

**Affiliations:** 1 Postgraduation School in Radiodiagnostics, Università degli Studi di Milano, Via Festa del Perdono 7, 20122 Milano, Italy; 2 Department of Biomedical Sciences for Health, Università degli Studi di Milano, Via Mangiagalli 31, 20133 Milano, Italy; 3 Unit of Radiology, ASST Nord Milano, Viale Matteotti 83, 20099 Sesto San Giovanni, Italy; 4 Postgraduation School in Cardiology, Università degli Studi di Milano, Via Festa del Perdono 7, 20122 Milano, Italy; 5 Pediatric and Adult Congenital Heart Centre, IRCCS Policlinico San Donato, Via Morandi 30, 20097 San Donato Milanese, Italy; 6 Department of Radiology, IRCCS Policlinico San Donato, Via Morandi 30, 20097 San Donato Milanese, Italy

**Keywords:** Tetralogy of Fallot, Gadolinium, Heart ventricles, Pulmonary valve, Magnetic resonance imaging

## Abstract

**Background:**

Our purpose was to evaluate the correlations between right ventricular (RV) late gadolinium enhancement (LGE) at cardiac magnetic resonance (CMR) in patients with tetralogy of Fallot (ToF) scheduled for pulmonary valve replacement (PVR) and post-PVR functional data.

**Methods:**

We retrospectively reviewed ToF patients scheduled for PVR who underwent two CMR examinations at our institution, one before the procedure (CMR-0), including contrast-enhanced sequences, and one after the procedure (CMR-1). Functional left and RV data were obtained by segmenting short-axis stacks on both CMR examinations, and normalised variations were calculated by dividing differences between CMR-1 and CMR-0 by the intercurring time interval, whereas the RV scar burden was assessed on CMR-0 LGE sequences both semiquantitatively and quantitatively. Data were reported as median and interquartile range, differences were appraised with the Mann–Whitney *U* test, while correlations were assessed with Spearman’s *ρ*.

**Results:**

Fifteen patients with a median age of 25 years (16–29), including 9 (60%) males, with a median time interval between CMR-0 and CMR-1 of 17 months (12–23), were retrospectively reviewed. The semiquantitative LGE score at CMR-0 was 7 (6–9), and LGE volume was 4.49 mL (3.70–5.78), covering 5.63% (4.92–7.00) of the RV. RV LGE score showed a moderate positive correlation with the normalised variation of RV stroke volume (*ρ* = 0.662, *p* = 0.007) and a borderline moderate positive correlation with the normalised variation of RV end-diastolic indexed volume (*ρ* = 0.513, *p* = 0.050).

**Conclusions:**

The assessment of RV LGE before PVR may provide insights on post-PVR functional data, potentially facilitating a patient-tailored treatment pathway.

## Key points

- Tetralogy of Fallot (ToF) patients undergo pulmonary valve replacement (PVR), reducing right ventricle (RV) stress.
- Late gadolinium enhancement (LGE) extent in PVR ToF patients predicts worse post-interventional RV volumetric changes.
- Assessing LGE before PVR may help predict functional parameters and plan a more tailored patient care.

## Introduction

Tetralogy of Fallot (ToF) is the most common cyanotic heart defect, with an estimated prevalence of 3 cases every 10,000 live births [1]. Nonetheless, the prevalence in adult population is increasing up to 1 in every 3,500 due to a combination of more accurate prenatal diagnoses and better surgical and follow-up options [2]. Therefore, tailored clinical pathways are warranted to ensure the best treatment standards in this expanding population.

Although valve-sparing techniques are encouraged for surgical repair, transannular patch is commonly needed for the relief of outflow obstruction, leading to free pulmonary regurgitation and chronic volume right ventricle (RV) overload [3]. Current guidelines indicate specific timings for pulmonary valve replacement (PVR) based on RV data, to revert ventricular remodelling and achieve the best outcomes [4].

Cardiac magnetic resonance (CMR) is a pivotal tool in the follow-up of this population, being the standard of care for the functional assessment of the RV [5, 6]. Along with ventricular morphology and function, contrast-enhanced CMR allows the assessment of focal myocardial fibrosis through late gadolinium enhancement (LGE). In ToF patients, fibrotic myocardial areas may be a result of ventricular remodelling and/or subsequent corrective surgical procedures [7]. Indeed, the assessment of LGE may yield a predictive value concerning ventricular functional parameters and clinical adverse events as ventricular arrythmias [8]. A previous work by Babu-Narayan et al. [9] proposed a semiquantitative scoring system for RV LGE that displayed significant correlations to clinical variables in ToF patients, being related to the onset of ventricular tachycardia [10]. Moreover, LGE has been identified as a predictor of left ventricular (LV) function in patients undergoing aortic valve replacement, with patients presenting a greater fibrosis extent benefitting less from surgical intervention [11].

Therefore, the aim of the present study was to evaluate LGE in ToF patients undergoing PVR and to assess its potential correlations with post-PVR CMR data.

## Material and methods

The local ethics committee (Ethics Committee of IRCCS Ospedale San Raffaele) approved this retrospective study (protocol code “CardioRetro”, number 122/int/2017; approved on September 14th, 2017, and amended on July 19th, 2022), and informed consent was waived due to the retrospective nature of the study.

### Study population

We retrospectively included all repaired ToF patients who underwent at least two CMR examinations (CMR-0 and CMR-1) at our institution and who underwent PVR during the intercurrent time interval, between March 2014 and December 2020. We subsequently excluded all those who did not undergo LGE evaluation at the first CMR examination.

### Image acquisition

All patients underwent CMR examinations acquired on a 1.5-T unit (MAGNETOM Aera, Siemens Healthineers, Erlangen, Germany) with a gradient power of 45 mT/m. All examinations were performed using a 48-channel surface-phased-array coil which was placed over patients’ chests while lying supine. Each acquisition included the sequences reported in Table 1, with LGE acquired after intravenous administration of 0.10 to 0.15 mmol/kg of gadobutrol (Gadovist, Bayer Healthcare, Leverkusen, Germany).

**Table 1:** Technical parameters for the main sequences used for the acquisition of cardiac magnetic resonance examinations in the study population

	**Cine**	**LGE**
**Sequence**	Steady-state-free precession	Inversion recovery turbo FLASH
**Time of echo (ms)**	1.7	3.3
**Time of repetition (ms)**	3.0	3.5
**Flip angle (°)**	60–80	25
**Slice thickness (mm)**	8	8
**Inversion time (ms)**	-	280
**Number of excitations**	1	1
**FoV read (mm)**	350	400
**FoV phase (%)**	81.3	68.8
**Phase oversampling (%)**	54	50
**PAT mode**	GRAPPA	GRAPPA

*FoV* Field of view, *LGE* Late gadolinium enhancement, *PAT* Parallel acquisition techniques

### Image analysis

For each CMR examination, short-axis cine sequences were segmented as part of the usual clinical workflow, by a radiologist (F.Se.) with 12 years of experience in cardiovascular imaging, for the assessment of LV and RV indexed end-diastolic volume (EDVi), indexed end-systolic volume (ESVi), stroke volume (SV), and ejection fraction (EF), with the Medis Suite MR Software (Medis Medical Imaging System bv., Leiden, the Netherlands).

**Fig. 1:**
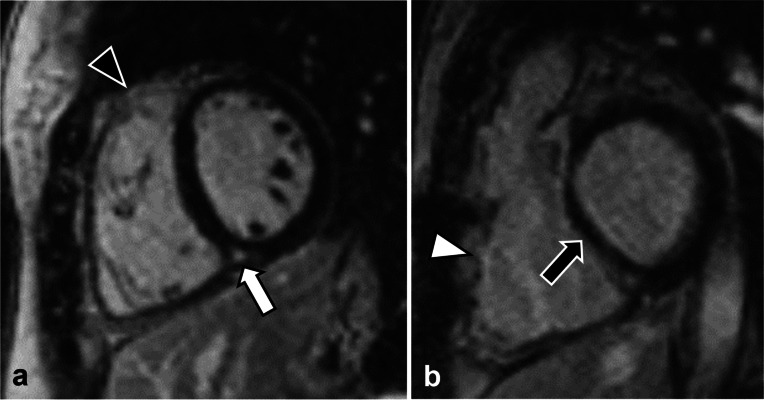
Example of semiquantitative late gadolinium enhancement scoring in a 29-year-old female tetralogy of Fallot patient before pulmonary valve replacement, who was assigned a score of 2 for the free wall of the right ventricle outflow tract (**a**, black arrowhead), 2 for the free wall of the right ventricle (**b**, white arrowhead), 2 for the right ventricular septal wall (**b**, black arrow), and 1 for the right ventricular insertion points (**a**, white arrow)

One radiologist (G.L.) with 4 years of experience in cardiovascular imaging reviewed all CMR datasets on the local picture archiving and communication system and scored all LGE sequences both according to the semiquantitative scoring system proposed by Babu-Narayan et al. [9] and quantitatively by segmenting short-axis LGE stacks on the Medis Suite MR software (QMass module); for such quantitative, semiautomatic segmentation, a threshold of 3 standard deviations, was chosen, according to previous works [12], and automatically segmented contours were subsequently manually edited. An example of the proposed scoring is shown in Fig. 1.

### Statistical analysis

Due to the expected paucity of the sample size, data were reported as median and interquartile range (IQR) and analysed via nonparametric statistics. To normalise data according to the different follow-up intervals between the first and second CMR examination among patients, variations in CMR parameters were divided by the intercurring time. Differences between the two CMR examinations were appraised with the Mann–Whitney *U*-test, while correlations were assessed with Spearman’s *ρ* and interpreted according to the framework proposed by Evans [13]. Statistical analyses were performed with Python v7 7.18.1. All *p*-values < 0.050 were considered as indicative of statistical significance [14]. For the three correlations with different LGE estimates assessed for each functional parameter, Bonferroni’s correction was applied to correlation analyses [15], leading to a threshold of *p*-value < 0.017.

## Results

### Study population

Out of 294 individual patients with ToF who underwent CMR at our institution, 48 had CMR examinations performed both before and after PVR, and, among them, 15 had undergone contrast-enhanced CMR before PVR. Hence, our final study population was composed by 15 patients who underwent PVR between two consecutive CMR examinations, 9 (60%) of whom males, with a median age of 25 years (IQR 16–29 years), acquired at a median interval of 17 months (IQR 12–23 months). The median interval between CMR-0 and PVR was 6 months (IQR 0–11 months), whereas the median interval between PVR and CMR-1 was 7 months (IQR 7–10 months).

Functional parameters from the LV and RV at the two CMR examinations are reported in Table 2, along with data concerning variations over time. Between CMR-0 and CMR-1, LV EDVi increased significantly (*p* = 0.025) from 70 mL/m^2^ (IQR 61–72 mL/m^2^) to 81 mL/m^2^ (IQR 69–92 mL/m^2^), and LV ESVi increased with borderline significance (*p* = 0.050) from 24 mL/m^2^ (IQR 21–28 mL/m^2^) to 32 mL/m^2^ (IQR 25–35 mL/m^2^), while RV EDVi decreased significantly (*p* = 0.009) from 120 mL/m^2^ (IQR 100–128 mL/m^2^) to 94 mL/m^2^ (IQR 82–101 mL/m^2^), and RV ESVi decreased significantly (*p* = 0.048) from 53 mL/m^2^ (IQR 41–61 mL/m^2^) to 42 mL/m^2^ (IQR 34–48 mL/m^2^).

**Table 2:** Demographics and clinical variables of the study population, including variations over time normalised for the time difference between CMR-0 and CMR-1 in months

***N***			15	
**Age (years)**			25 (16–29)	
**Males (%)**			9 (60)	
**Follow-up (months)**			17 (12–23)	
	**CMR-0**	**CMR-1**	**Variation per month**	**p**_**CMR-0−CMR-1**_
**LV EDVi (mL/m**^**2**^**)**	70 (61–72)	81 (69–92)	0 (0–1)	0.025^a^
**LV ESVi (mL/m**^**2**^**)**	24 (21–28)	32 (25–35)	0 (0–1)	0.050^a^
**LV SV (mL)**	67 (63–77)	82 (68–92)	1 (0–1)	0.060
**LV EF (%)**	64 (59–68)	62 (59–65)	0 (-1–0)	0.240
**RV EDVi (mL/m**^**2**^**)**	120 (100–128)	94 (82–101)	-1 (-2–0)	0.009^a^
**RV ESVi (mL/m**^**2**^**)**	53 (41–61)	42 (34–48)	-1 (-1–0)	0.048^a^
**RV SV (mL)**	124 (102–124)	88 (66–92)	-2 (-2–-1)	0.017^a^
**RV EF (%)**	53 (51–59)	53 (50–60)	0 (0–1)	0.339
**RV LGE (mL)**	4.49 (3.70–5.78)	−		
**RV LGE (%)**	5.63 (4.92–7.00)	−		
**RV LGE score**	7 (6–9)	−		

*CMR-0* First cardiac magnetic resonance examination, *CMR-1* Follow-up cardiac magnetic resonance examination, after pulmonary valve replacement, *LV* Left ventricle, *RV* Right ventricle, *EDVi* Indexed end-diastolic volume, *ESVi* Indexed end-systolic volume, *SV* Stroke volume, *EF* Ejection fraction

^a^Denotes statistical significance

### Late gadolinium enhancement

**Fig. 2:**
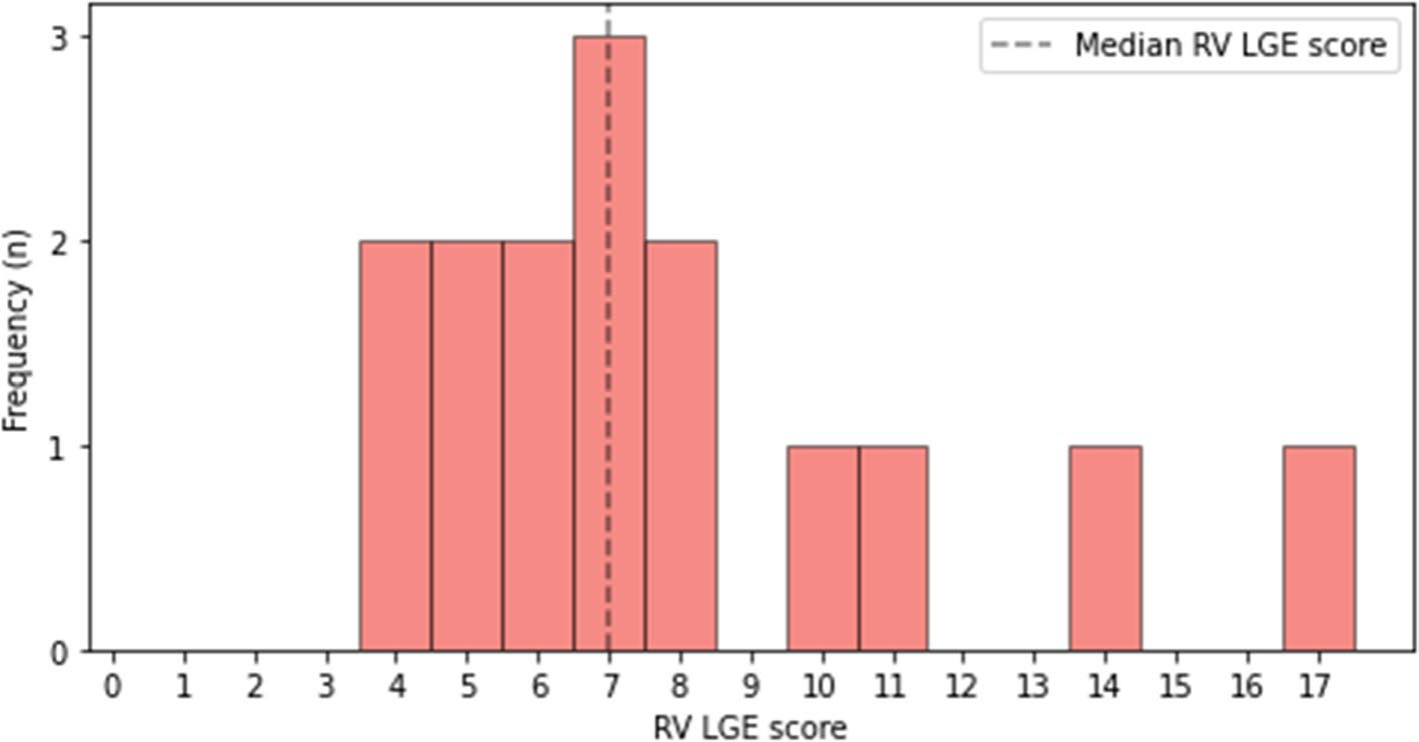
Distribution of the right ventricular (RV) late gadolinium enhancement (LGE) score as proposed by Babu-Narayan et al. [9] among the study population

All 15 patients presented some degree of LGE. The median RV LGE score of our patients at CMR-0 was 7 (IQR 6–9), while the median absolute extent of LGE was 4.49 mL (IQR 3.70–5.78 mL), accounting for 5.63% (IQR 4.92–7.00%) of the RV volume. The distribution of RV LGE scores is reported in Fig. 2. LGE data from the two CMR examinations are reported in Table 2. While LGE extent correlated with LGE percentage (*ρ* = 0.550, *p* = 0.034), there were no significant correlations between RV LGE score and LGE extent (*ρ* = 0.099, *p* = 0.726) or percentage (*ρ* = 0.273, *p* = 0.324). Moreover, RV LGE score displayed a strong negative correlation with RV SV (*ρ* =  -0.609, *p* = 0.016). Correlations between LGE at the CMR-0 and functional data at CMR-1 are reported in Table 3.

**Table 3:** Correlations between extent, percentage, and score of right ventricular (RV) late gadolinium enhancement (LGE) and functional parameters

		**RV LGE (mL)**	**RV LGE (%)**	**RV LGE score**
**CMR-0**	**LV EDVi (mL/m**^**2**^**)**	*ρ* = 0.331, *p* = 0.227	*ρ* = 0.044, *p* = 0.874	*ρ* = -0.119, *p* = 0.672
	**LV ESVi (mL/m**^**2**^**)**	*ρ* = 0.303, *p* = 0.272	*ρ* = -0.066, *p* = 0.814	*ρ* = -0.128, *p* = 0.648
	**LV SV (mL)**	*ρ* = 0.327, *p* = 0.234	*ρ* = -0.039, *p* = 0.889	*ρ* = -0.387, *p* = 0.154
	**LV EF (%)**	*ρ* = 0.286, *p* = 0.302	*ρ* = 0.004, *p* = 0.990	*ρ* = 0.072, *p* = 0.799
	**RV EDVi (mL/m**^**2**^**)**	*ρ* = 0.225, *p* = 0.420	*ρ* = -0.250, *p* = 0.369	*ρ* = 0.020, *p* = 0.944
	**RV ESVi (mL/m**^**2**^**)**	*ρ* = 0.172, *p* = 0.541	*ρ* = -0.113, *p* = 0.689	*ρ* = 0.070, *p* = 0.804
	**RV SV (mL)**	*ρ* = 0.004, *p* = 0.990	*ρ* = -0.411, *p* = 0.128	***ρ* = -0.609, *p* = 0.016**^**a**^
	**RV EF (%)**	*ρ* = -0.308, *p* = 0.264	*ρ* = -0.204, *p* = 0.465	*ρ* = -0.287, *p* = 0.300
**CMR-1**	**LV EDVi (mL/m**^**2**^**)**	*ρ* = -0.004, *p* = 0.990	*ρ* = -0.263, *p* = 0.344	*ρ* = 0.120, *p* = 0.671
	**LV ESVi (mL/m**^**2**^**)**	*ρ* = -0.059, *p* = 0.835	*ρ* = -0.298, *p* = 0.280	*ρ* = 0.183, *p* = 0.515
	**LV SV (mL)**	*ρ* = 0.239, *p* = 0.390	*ρ* = -0.332, *p* = 0.226	*ρ* = -0.353, *p* = 0.197
	**LV EF (%)**	*ρ* = -0.117, *p* = 0.679	*ρ* = -0.147, *p* = 0.601	***ρ* = -0.517, *p* = 0.048**^**b**^
	**RV EDVi (mL/m**^**2**^**)**	*ρ* = 0.136, *p* = 0.629	*ρ* = 0.102, *p* = 0.717	*ρ* = 0.088, *p* = 0.754
	**RV ESVi (mL/m**^**2**^**)**	*ρ* = 0.184, *p* = 0.511	*ρ* = 0.243, *p* = 0.383	*ρ* = 0.221, *p* = 0.428
	**RV SV (mL)**	*ρ* = 0.408, *p* = 0.132	*ρ* = -0.191, *p* = 0.495	*ρ* = -0.437, *p* = 0.103
	**RV EF (%)**	*ρ* = -0.187, *p* = 0.505	*ρ* = -0.240, 0 = 0.388	***ρ* = -0.524, *p* = 0.045**^**b**^
**Variation per month**	***Δ*LV EDVi (mL/m**^**2**^**)**	*ρ* = -0.457, *p* = 0.087	*ρ* = -0.250, *p* = 0.369	*ρ* = 0.200, *p* = 0.476
	***Δ*LV ESVi (mL/m**^**2**^**)**	***ρ* = ** **-0.557, *p* = 0.031**^**a**^	*ρ* = -0.289, *p* = 0.296	*ρ* = 0.392, *p* = 0.148
	***Δ*LV SV (mL)**	*ρ* = -0.168, *p* = 0.550	*ρ* = -0.311, *p* = 0.260	*ρ* = 0.115, *p* = 0.683
	***Δ*LV EF (%)**	*ρ* = 0.289, *p* = 0.296	*ρ* = -0.025, *p* = 0.930	*ρ* = -0.156, *p* = 0.578
	***Δ*RV EDVi (mL/m**^**2**^**)**	*ρ* = 0.100, *p* = 0.723	*ρ* = 0.447, *p* = 0.095	***ρ* = 0.513, *p* = 0.051**^**b**^
	***Δ*RV ESVi (mL/m**^**2**^**)**	*ρ* = -0.257, *p* = 0.355	*ρ* = 0.232, *p* = 0.405	*ρ* = 0.259, *p* = 0.351
	***Δ*RV SV (mL)**	*ρ* = 0.159, *p* = 0.571	*ρ* = 0.324, *p* = 0.240	***ρ* = 0.662, *p* = 0.007**^**a**^
	***Δ*RV EF (%)**	*ρ* = 0.472, *p* = 0.076	*ρ* = 0.141, *p* = 0.616	*ρ* = 0.034, *p* = 0.904

*CMR-0* First cardiac magnetic resonance examination, *CMR-1* Follow-up cardiac magnetic resonance examination, after pulmonary valve replacement, *LV* Left ventricle, *RV* Right ventricle, *EDVi* Indexed end-diastolic volume, *ESVi* Indexed end-systolic volume, *SV* Stroke volume, *EF* Ejection fraction

^a^Denotes statistical significance, while ^b^denotes borderline statistical significance

### Correlation of late gadolinium enhancement and follow-up functional parameters

**Fig. 3:**
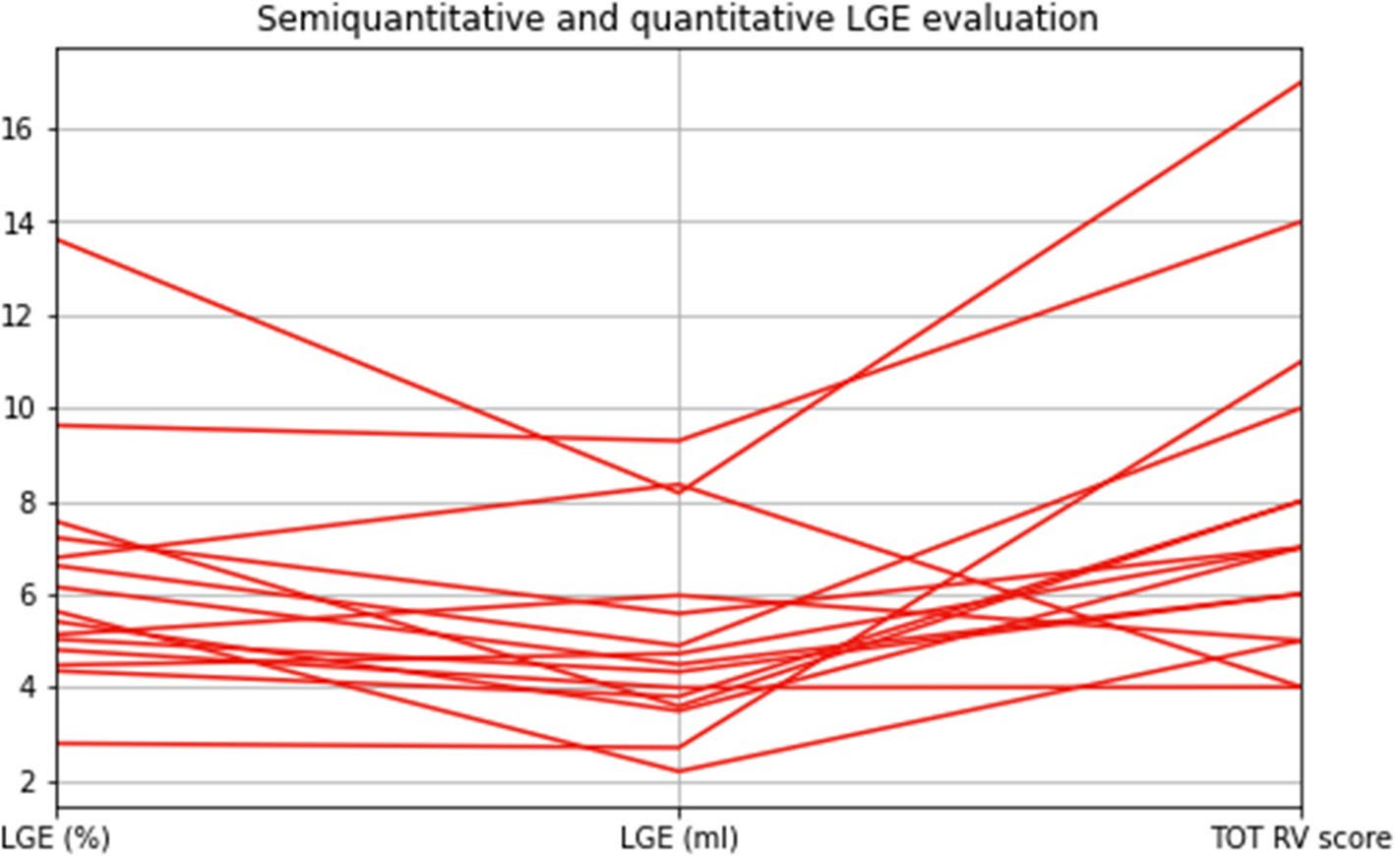
Relations among right ventricular (RV) late gadolinium enhancement (LGE) quantitative and qualitative estimates, represented by percentage over the left ventricle (%), volume (mL), and the total LGE score (TOT RV score) as proposed by Babu-Narayan et al. [9]

**Fig. 4:**
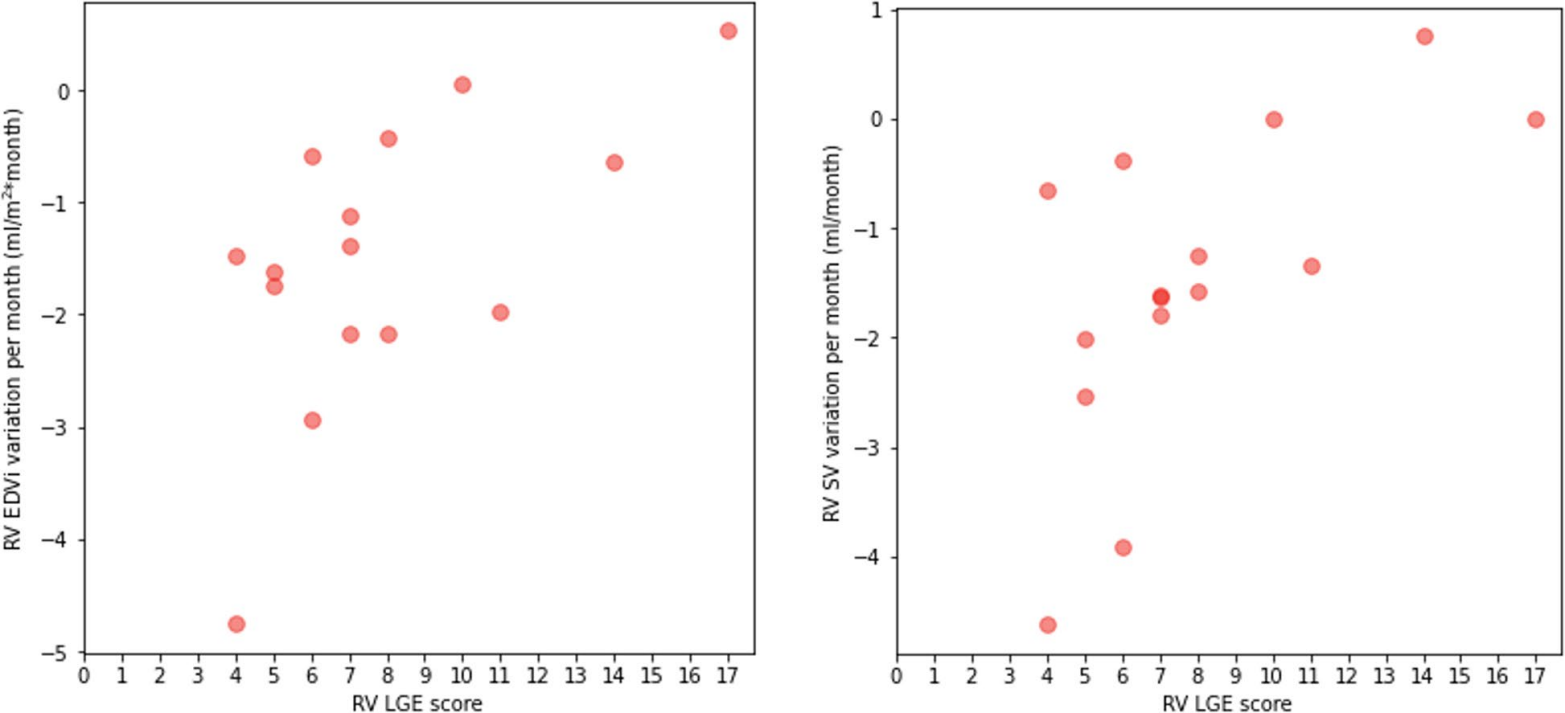
Scatterplots depicting the correlations between right ventricular (RV) late gadolinium enhancement (LGE) score and the monthly variation of RV end-diastolic indexed volume (EDVi) and RV stroke volume (SV), respectively

Concerning associations between LGE at CMR-0, and normalised variations of functional parameters, RV LGE volume and extent did not yield any significant correlation with any normalised variation data. Relations between LGE results are shown in Fig. 3. Conversely, RV LGE score showed a moderate positive correlation with the normalised variation of RV SV (*ρ* = 0.662, *p* = 0.007), and a moderate positive, even though not significant, correlation with the normalised variation of RV EDVi (*ρ* = 0.513, *p* = 0.051), as depicted in Fig. 4. All correlations between LGE parameters at CMR-0 and functional data at CMR-1 are displayed in Table 3.

## Discussion

The main findings of this study include the correlations between pre-procedural RV scarring in patients referred for PVR and post-PVR variations of functional cardiac parameters, suggesting that a higher extent of myocardial scar might be a predictor of worse ventricular remodelling.

To the best of our knowledge, this is among the first works investigating the predictive role of contrast-enhanced CMR LGE in ToF patients referred for PVR. Our patient population for this study is well-representative of young adult ToF patients referred for PVR, with a prevalence of males and a median age of 25 years [16]. Overall, RV indexed volumes in our cohort were in line with the limits reported in the indications for PVR to achieve the best outcomes in terms of functional data [17].

Concerning functional data, the increase of LV EDVi and ESVi after PVR may be due to a decrease in the volume and pressure overload on the RV which is transmitted to the LV via the interventricular septum, effectively allowing the LV to improve its expansion [18, 19]. Additionally, the decrease observed in RV EDVi, ESVi, and SV was an expected positive consequence of PVR, and the regression towards normal RV volumes may suggest in our cohort an adequate intervention timing [20, 21].

Regarding the RV, the median RV LGE score of our population was higher than that reported by Babu-Narayan et al. [9] in a group of adult ToF patients with an average age of 32 years and by Spiewak et al. [22] in a ToF cohort with an average age of 26 years. Similar differences may also be observed for the median absolute extent and percentage of RV LGE. This could be due to the fact that our population only comprised ToF patients scheduled to undergo PVR, who could thus present with a worse overall myocardial condition, resulting in larger scarring.

The lack of correlation between RV LGE score and LGE extent is most likely due to two main causes. First, the semiquantitative score is established evaluating both long-axis and short-axis scans, whereas the quantitative score may only be calculated on short-axis stacks. Hence, some areas which are not included in the latter are instead considered by the visual scoring system. Second, a visual semiquantitative score may not necessarily reflect the overall ventricular scar burden, especially considering that discrete areas are evaluated [23].

The positive correlations observed between RV LGE score at CMR-0 and normalised, per-month, variations of RV EDVi and RV SV in patients undergoing PVR suggest that a higher pre-PVR fibrosis results into a lower benefit from intervention, with a smaller reduction in diastolic RV remodelling and resulting SV. Indeed, myocardial fibrosis observed through LGE represents areas which might be less plastic and less adaptable to remodelling; thus, a greater scar extent might result in a lesser ventricular plasticity [24]. Although there was a trend towards post-PVR RV volumes normalisation even in the presence of a relatively high fibrotic burden, our results suggest that higher pre-PVR fibrosis predicts a lower benefit from intervention.

Despite the potential role of LGE in predicting a worse myocardial function in ToF patients, gadolinium-based contrast agents (GBCAs) still pose some issues, such as gadolinium brain depositions [25]. Concerns are anyway counteracted by the absence of reported clinical effects of these deposits [26]. Regarding the acute effects of GBCAs, in a recent study, the overall rate of severe, life-threatening, adverse events related to the GBCA administration in cardiac MRI examinations was well below 1% [27].

The role of GBCAs in supporting diagnosis in CMR, even in ToF patients, is relevant, having displayed significant relations to functional parameters and clinical adverse events. However, the assessment of LGE during every CMR examination ToF patients undergo may not be necessary, as a recent study by Saengsin et al. [28] showed how variations in myocardial scarring are trivial during short time frames. Hence, limiting the use of GBCAs to selected cases, such as in ToF patients scheduled to undergo PVR, may be reasonable. In this way, volumetric parameters could be predicted from a pre-PVR CMR examination, allowing a better stratification of patients scheduled to undergo PVR, and future developments could potentially include an even more accurate delineation of appropriate interventional timings.

Our study presents some limitations. First, the size of the study sample is relatively small, pertaining only to one single institution, and thus, potential correlations between LGE and other functional parameters at either time point or their variation might be missed for such reason. Indeed, ToF is a relatively rare pathology (3 cases every 10,000 live births [1]), and, in our retrospective analysis, ToF patients undergoing contrast-enhanced CMR before, and CMR after PVR, were 15 out of 294. Nevertheless, evidence concerning the potential predictive role of RV LGE score on functional parameters appears present even in spite of the paucity of the study population. In this sense, future developments could include detecting whether PVR or the presence of LGE yields a higher impact on RV functional parameters. Additionally, as LGE is only performed according to clinical needs, and not in a routine fashion, our results would need to be confirmed on larger, more heterogeneous populations. Second, we did not collect clinical data pertaining to potential adverse events or complications occurring in our study population after PVR; thus, we could not assess the correlations between LGE and such events. However, previous literature shows that presenting with higher RV EDVi may lead to a higher prevalence of adverse events [29], thus possibly inducting a higher pre-PVR LGE among risk factors. Third, time intervals between CMR examinations were not standardised; therefore, we had to assess normalised variations of functional parameters to detect a potential predictive role of pre-PVR LGE. However, despite the noteworthy heterogeneity, we were still able to appraise improvements in RV volumes and function after PVR, thus indicating that the post-PVR follow-up timing was adequate to detect variations in functional parameters. Fourth, we did not include an evaluation of T1 mapping in our population, as T1 mapping sequences were not acquired in most of our patients due to time constraints. Additionally, T1 mapping of the RV, which could be of greater interest in our population compared to that of the LV, implies some intrinsic technical challenges, which may render its results less accurate [30, 31]. Lastly, while patients did receive different doses of contrast agents as imaging protocols changed through the years, we may expect this issue to have a limited effect on LGE assessment. Indeed, previous studies showed that different contrast doses still yield similar image quality, therefore suggesting that LGE segmentation with manual corrections would still be viable [32].

In conclusion, the assessment of RV LGE burden may provide additional insight for a more patient-tailored and personalised management of ToF patients referred for PVR, displaying a correlation to post-procedural functional data. Given the correlations between LGE extent and clinical outcomes and adverse events, in view of its limited variability over time, it may be suggested that RV LGE should be assessed at least at certain timepoints during the course of ToF patients CMR follow-up, possibly before undergoing PVR, as a potential part of pre-procedural planning.

## Data Availability

The datasets used and/or analysed during the current study are available from the corresponding author on reasonable request.

## Abbreviations

CMR: Cardiac magnetic resonance
EDVi: End-diastolic volume index
EF: Ejection fraction
ESVi: End-systolic volume index
GBCAs: Gadolinium-based contrast agents
IQR: Interquartile range
LGE: Late gadolinium enhancement
LV: Left ventricle
PVR: Pulmonary valve replacement
RV: Right ventricle
SV: Stroke volume
ToF: Tetralogy of Fallot
